# In vitro testing of shear bond strength of orthodontic brackets bonded to different novel CAD/CAM ceramics

**DOI:** 10.34172/joddd.2020.042

**Published:** 2020-10-24

**Authors:** Eglal Ahmed Ghozy, Marwa Sameh Shamaa, Ahmed A. El-Bialy

**Affiliations:** ^1^Department of Orthodontics, Faculty of Dentistry, Mansoura University, Egypt

**Keywords:** Bond strength, CAD/CAM, Orthodontic brackets

## Abstract

**Background.** The present study aimed to evaluate the bond strength of metal bracket (MB) and ceramic bracket (CB) bonded to different CAD/CAM ceramic substrates etched with hydrofluoric acid (HFA) vs. phosphoric acid (PA).

**Methods.** A total of 120 CAD/CAM ceramic blocks in 12 groups were fabricated from three different CAD/CAM ceramic materials: VITABLOCS Mark II, VITAENAMIC, and IPS e.max CAD. Each ceramic material group was divided into two etching groups: one treated with 9.5% HFA and the other treated with 37%. Sixty metal and CBs of the upper right central incisor were bonded to the HFA-treated blocks. Another 60 metal and CBs were bonded to the PA treated blocks. All the bonded specimens were thermocycled before shear bond strength (SBS) testing. Then the bond failure mode was recorded

**Results.** There were no significant differences in SBS values between the three CAD/CAM ceramic materials. The HFA-treated specimens exhibited significantly higher SBS values than the PA-treated specimens. Also, the SBS values of CBs were significantly higher than the metal brackets (MBs). The adhesive remnant index (ARI) score was 4 for most of the groups, indicating that almost no adhesive remained on the porcelain surface.

**Conclusion.** The CAD/CAM ceramic type did not influence SBS; however, HFA exhibited significantly higher SBS compared to PA.

## Introduction

The use of all-ceramic crowns has increased dramatically due to the increased demand for esthetic restorations. With the increasing number of adults seeking orthodontic therapy, orthodontists face the challenge of bonding to different types of all-ceramic materials as bonding of orthodontic brackets to these materials differs from bonding to the enamel surface.^1^

The bonding of orthodontic brackets to ceramics can be affected by many factors, including the type of porcelain, surface conditioning method, the bracket material, retention mode (bracket base), the adhesive properties, the light-curing source, storage time, thermocycling, debonding force, and the clinician’s skill.^2,3^

As the porcelain structure is inert, several surface treatment methods have been tried to enhance the bond strength of orthodontic attachments to the ceramic surface.^2^ These methods could be mechanical or chemical or a combination of both.^4^

Mechanical methods include sandblasting or using a coarse diamond stone.^5-7^ Although these methods significantly increase the bond strength, they can increase the probability of porcelain fracture on debonding.^8^

Chemical methods are implemented by etching with hydrofluoric acid (HFA) gel, phosphoric acid (PA) gel, or altering the porcelain bonding affinity to adhesive materials by using a silane coupling agent.^5-10^ The most widely used ceramic acid etching agent is a 9.6% HFA gel.^4^ Since HFA is a very strong acid, it should be handled with great care to avoid any contact with the soft tissues.^5,9,11,12^ On the other hand, treating porcelain surfaces with 37% PA was documented to produce adequate bond strength that is clinically accepted compared with that produced by HFA.^9,11^ The silane reacts with the silica within the porcelain and the adhesive resin’s organic groups, creating a bond between the two materials and enhancing the bond strength to porcelain surfaces.^4^

CAD/CAM blocks of conventional feldspathic silicate ceramic, lithium disilicate glass-ceramic, and polymer-infiltrated ceramic network (hybrid ceramic) are different from each other in their formulations. Feldspathic silicate and lithium disilicate glass ceramics are composed mainly of a mixture of feldspathic crystalline or lithium disilicate particles set in a glassy background. On the other hand, hybrid ceramics are composed of a copolymer (urethane dimethacrylate and triethylene glycol dimethacrylate), which infiltrate the porous feldspathic ceramic matrix.^13-15^ These differences can affect the bond strength of orthodontic brackets bonded to them.^1^ To the best of our knowledge, no previous study has compared different etching acids applied to these three different CAD/CAM ceramic materials and their effect on bonding both metal and ceramic orthodontic brackets.

This study evaluated the shear bond strength** (**SBS) values of metal bracket (MB) and ceramic bracket (CB) bonded to different CAD/CAM ceramic materials etched with HFA vs. PA and determined the bond failure mode.

## Methods

A total of 120 CAD/CAM ceramic blocks in 12 groups (n=10) were fabricated from three different CAD/CAM ceramic materials: Vitablocs Mark II (VM) (Vita, Bad Säckingen, Germany), Vita Enamic (VE) (Vita, Bad Säckingen, Germany), and IPS e.max CAD (EM) (Ivoclar Vivadent AG, Liechtenstein). The disks were fabricated with 14×12×2-mm dimensions using a low-speed cutting machine (IsoMet 4000 micro-saw, Buehler, USA). The disks were then glazed, each according to its manufacturer’s instructions.

Each ceramic material group was divided into two etching subgroups: treated with 9.5% HFA (Yellow Porcelain Etch, Cerkamed, Stalowa Wola, Poland) and treated with 37% PA (Eco-Etch Etching Gel, Ivoclar Vivadent, NY, USA). Sixty metal and ceramic orthodontic brackets of the upper right central incisor (Ortho-DIRECT, USA) were bonded to the HFA-treated blocks. Another sixty metal and CBs were bonded to the PA-treated blocks in the study’s twelve groups, as shown in Table 1.

**Table 1:** Groups of this study

**Group number**	**Details**		
	**CAD/CAM** **ceramic**	**Acid etch**	**Bracket material**
1	VM	HFA	MB
2	VM	HFA	CB
3	VM	PA	MB
4	VM	PA	CB
5	VE	HFA	MB
6	VE	HFA	CB
7	VE	PA	MB
8	VE	PA	CB
9	EM	HFA	MB
10	EM	HFA	CB
11	EM	PA	MB
12	EM	PA	CB

The specimens in groups 1, 2, 5, 6, 9, and 10 were conditioned with 9.5% HFA for 1 minute, rinsed for 1 minute, and then air-dried. While the specimens in groups 3, 4, 7, 8, 11, and 12 were conditioned with 37% PA for 1 minute, rinsed for 1 minute, and then air-dried. Afterward, a single coat of silane (SILAN, Cerkamed, Stalowa Wola, Poland) was applied and allowed to dry; TransbondXT primer (3M Unitek, CA, USA) was then applied and air-thinned.

MBs in groups 1, 3, 5, 7, 9, and 11 and CBs in groups 2, 4, 6, 8, 10, and 12 were then bonded using Transbond XT adhesive paste (3M Unitek, CA, USA) and pressed hard against the middle of the ceramic surface. The excess adhesive was removed from all around the bracket base, followed by light-curing using LiteQ LD-107 light-curing unit (Monitex, New Taipei City, Taiwan).

Following the bonding procedures, all the specimens were stored in distilled water for 24 hours and then thermocycled for 1000 cycles in hot and cold baths at 5‒55±4°C for 30 seconds. As a means of artificial aging, a dual-interval procedure was performed to simulate the oral environment before testing. This was carried out in the Dental Materials Department, Mansoura University.

**Figure 1:**
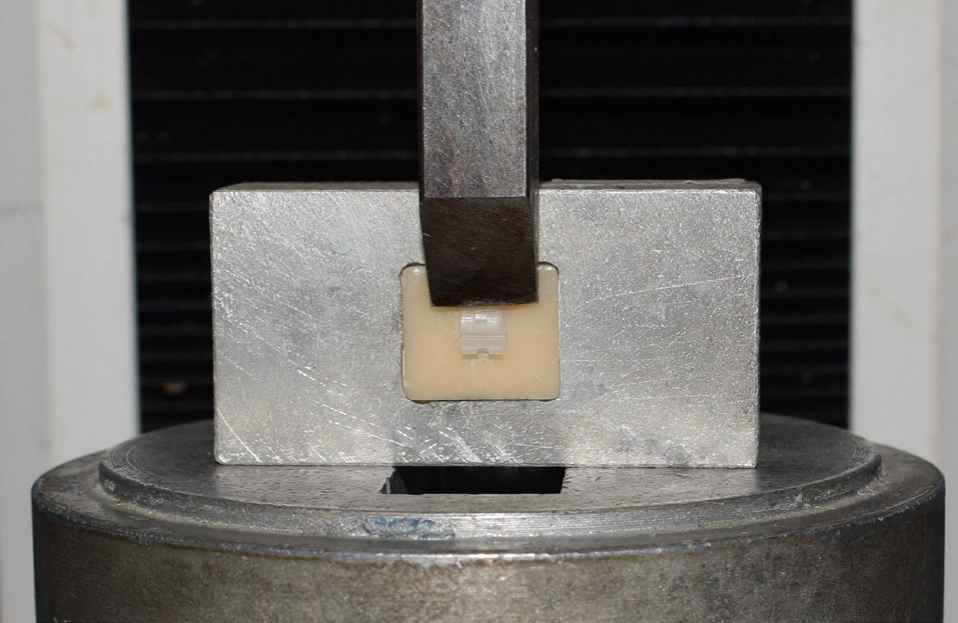
The chisel tip was positioned to touch only the base of the bracket.

The SBS was measured using an Instron universal testing machine (Model 3345; Norwood, USA) with the mono-beveled chisel attached to the upper movable compartment of the testing machine to apply a compressive loading on each specimen. The load was applied in the occlusogingival direction at a crosshead speed of 0.5 mm/min. The chisel tip was settled to touch only the bracket base, as shown in Figure 1. The maximum failure load was recorded in Newton (N). The maximum failure load was then divided by the bracket base surface area, measured using a digital caliper, to present the bond strength in MPa.

The specimens were evaluated under a stereomicroscope (Olympus, Tokyo, Japan) at ×20 magnification to determine the adhesive remnant index (ARI). The measurements were conducted, using scores from 1 to 5, as modified by Bishara et al.^16^

1: All the adhesive remains on the ceramic surface with the impression of the bracket base (100%).

2: >90% of the adhesive left on the ceramic surface (>90%).

3: <90% but >10% of the adhesive remaining on the surface (90-10%).

4: <10% of the adhesive remains on the ceramic surface (<10%).

5: No adhesive remains on the ceramic surface (0%).

Data were collected and analyzed using one-way ANOVA, Kruskal-Wallis H test, chi-square test, *t* test, Mann-Whitney U test, pairwise comparisons, and three-way ANOVA.

## Results

The descriptive statistics of SBS values are presented in Table 2. The highest mean SBS value was recorded in the VE group treated with HFA when CBs were bonded to them. On the other hand, the lowest mean SBS was recorded in the EM group treated with PA when MBs were bonded to them.

**Table 2:** Descriptive statistics of SBS in the 12 groups

**Group**	**Mean ± SD**	**SE**	**95% CI of the mean**	**Median (IQR)**	**Minimum-maximum**
G1	10.2±3	0.95	8.1–12.4	10.4 (8.9‒12.1)	3.6–14.7
G2	10.6±5.1	1.6	6.9–14.3	8.7 (6.9‒13.3)	6.5–20.8
G3	6.9±2.3	0.74	5.2–8.5	6.6 (6–8.4)	2.1–10.9
G4	8.9±4.6	1.4	5.7–12.2	8.1 (6.3–12.7)	0.69–17.1
G5	8.6±2.9	0.92	6.6–10.7	8.9 (6.8–11.2)	2.8–12.1
G6	10.9±4.8	1.5	7.5–4.3	10.6 (6.7–15.5)	4.5–18.4
G7	6.5±1.7	0.55	5.2–7.7	6.5 (5.2–7.2)	4–9.5
G8	9.5±3.8	1.2	6.8–12.2	10.1 (6.3–13.1)	3.2–13.5
G9	8.5±1.8	0.58	7.2–9.8	8.5 (6.7–10.2)	5.8–11.2
G10	9.4±4.3	1.4	6.3–12.5	9.3 (6.3–13.1)	3.1‒17.2
G11	6.2±1.8	0.57	4.95–7.5	6.2 (5.6–7.5)	2–8.5
G12	7.3±2.7	0.86	5.3–9.2	6.7 (5.9–7.9)	4.1–14.1

There were no significant differences in SBS values between the three CAD/CAM ceramic materials. However, there were significantly higher SBS values in HFA vs. PA. Also, there were significantly higher SBS values in CB vs. MB. There was no main effect of CAD/CAM ceramic material on SBS, and there was a significant main effect of etching method and bracket type on SBS. There was no significant interaction, as shown in Table 3.

**Table 3:** Comparison of mean SBS in MPa, standard deviations (SD), standard errors (SE) and 95% confidence of intervals (CI) of the different variables included in the study

**Variable**	**Type**	**Mean ± SD**	**SEM**	**95% CI**	***P*** ** value**
CAD/CAM material	VM (n=40)	9.2±4.1	0.64	7.9–10.5	0.255
	VE (n=40)	8.9±3.7	0.59	7.7–10.1	
	EM (n=40)	7.8±3	0.48	6.9–8.8	
Etching method	HFA (n=60)	9.7±3.8	0.49	8.7–10.7	<0.0005
	PA (n=60)	7.5±3.1	0.4	6.7–8.4	
Bracket type	MB (n=60)	7.8±2.6	0.34	7.1‒8.5	0.050
	CB (n=60)	9.4±4.3	0.55	8.3‒10.5	

**Figure 2:**
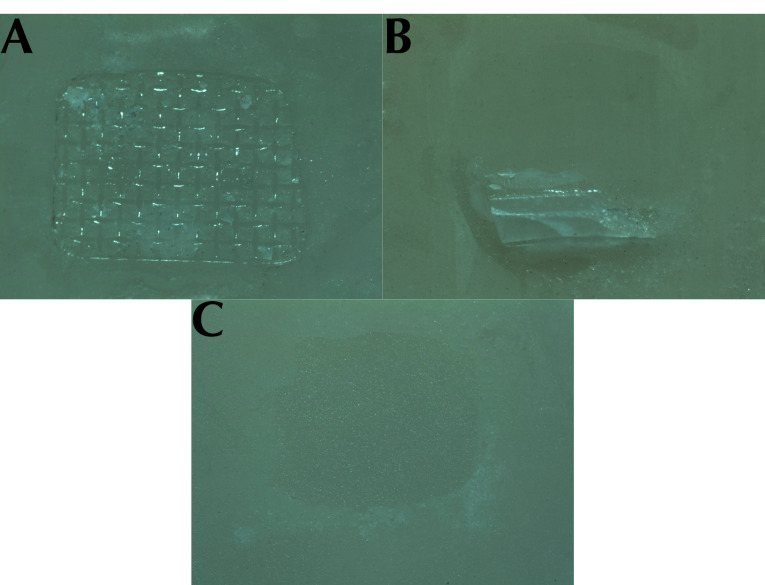
A specimen in **(A)** G1, (**B**) G8 and (**C**) G11 under stereomicroscope at ×20 magnifications.

There was a significant difference in ARI scores between the study groups (Figures 2A-C). The ARI scores are presented in Table 4.

**Table 4:** Comparison of ARI scores between the 12 study groups

**Group**	**ARI Score**				
	**1**	**2**	**3**	**4**	**5**
G1 (VM+HF+M)	4_a_	3_a_	1_a, b_	0_b_	2_a, b_
	30.8%	25.0%	11.1%	0.0%	5.9%
G2 (VM+HF+C)	2_a_	0_a_	0_a_	5_a_	3_a_
	15.4%	0.0%	0.0%	9.6%	8.8%
G3 (VM+PA+M)	0_a_	0_a_	0_a_	5_a_	5_a_
	0.0%	0.0%	0.0%	9.6%	14.7%
G4 (VM+PA+C)	0_a_	0_a_	0_a_	7_a_	3_a_
	0.0%	0.0%	0.0%	13.5%	8.8%
G5 (VE+HF+M)	3_a_	1_a_	1_a_	4_a_	1_a_
	23.1%	8.3%	11.1%	7.7%	2.9%
G6 (VE+HF+C)	0_a, b_	0_a, b_	3_b_	7_a, b_	0_a_
	0.0%	0.0%	33.3%	13.5%	0.0%
G7 (VE+PA+M)	0_a_	0_a_	0_a_	7_a_	3_a_
	0.0%	0.0%	0.0%	13.5%	8.8%
G8 (VE+PA+C)	0_a_	1_a_	0_a_	6_a_	3_a_
	0.0%	8.3%	0.0%	11.5%	8.8%
G9 (EM+HF+M)	3_a_	2_a_	2_a_	2_a_	1_a_
	23.1%	16.7%	22.2%	3.8%	2.9%
G10 (EM+HF+C)	1_a, b_	4_b_	1_a, b_	1_a_	3_a, b_
	7.7%	33.3%	11.1%	1.9%	8.8%
G11 (EM+PA+M)	0_a_	0_a_	0_a_	3_a_	7_a_
	0.0%	0.0%	0.0%	5.8%	20.6%
G12 (EM+PA+C)	0_a_	1_a_	1_a_	5_a_	3_a_
	0.0%	8.3%	11.1%	9.6%	8.8%

*Note*. Data are expressed as frequencies and percentages. *P* value by chi-square test. For comparison of column proportions with Bonferroni adjustment. similar letters = insignificant difference while different letters = significant difference. **χ** ^2^ **=** 84.422

## Discussion

The present study investigated the SBS of MB and CB bonded to different CAD/CAM materials: feldspathic porcelain (VM), hybrid ceramic of dual network structure of polymer-infiltrated feldspathic ceramic (VE), and lithium disilicate glass ceramic (EM), in which different etching protocols using HFA vs. PA were applied. Each group included 10 specimens as advocated to conduct SBS testing by Fox et al.^17^

At the end of orthodontic therapy, the brackets should be detached from the restoration surfaces, selectively without damaging the restorative material structure. That is why a high bond strength is not favored. The SBS values of 6‒8 MPa are sufficient for orthodontic attachments bonded onto tooth surfaces in clinical practice.^18^

In the present study, 49.2% of SBS values were above this optimum range, 32.5% were within this optimum range, and only 18.3% were below the optimum range.

The bonding of orthodontic brackets to ceramics can be affected by many factors, including the porcelain type, surface conditioning method, the bracket material, the retention mode (bracket base), the adhesive properties, the light-curing source, storage time, thermocycling, debonding force, and the clinician’s skill. ^2^

In the present study, no main effect of CAD/CAM ceramic material on SBS was found. However, there was a significant main effect of both etching method and bracket type on SBS. There was no statistically significant interaction.

These results were similar to a previous study by Dilber et al,^19^ who found that the average SBS values were significantly influenced by the substrate treatment protocol but not the CAD/CAM substrate category.

On the other hand, these results were relatively consistent with a previous study by Buyuk and Kucukekenci,^1^ who found that both CAD/CAM material types, i.e., feldspathic ceramic, resin nanoceramic, and hybrid ceramic, and adhesion protocols significantly affected the bond strength.On the other hand, the conditioning methods did not. These differences can be attributed to the different methodology followed in that study.

Also, Bilgic et al^20^ concluded that SBS of CB bonded to different ceramic substrates could exhibit different values due to the porcelain crown type.

The results showed no significant differences in SBS between the three CAD/CAM ceramic materials in the present study. The highest mean SBS value was recorded in VM groups (9.2±4.1 MPa), and the lowest was recorded in EM groups (7.8±3 MPa). The mean SBS value for VE groups was 8.9±3.7 MPa.

However, Buyuk and Kucukekenci^1^ found that^the^ highest SBS value was recorded in the hybrid ceramic group, conditioned with HFA and Transbond XT adhesive primer. Also, Türk et al^12^ found that lithium disilicate ceramic produced SBS values higher than those produced with feldspathic ceramic. Bilgic et al^20^ found that the SBS of CB bonded to different ceramic substrates could exhibit different values due to the porcelain crown type.

Calamia^21^ suggested using powerful acids such as 9.6% HFA for etching porcelain. However, HFA is severely corrosive and can severely injure soft tissues and damage the tooth structure.^22^ PA can be a safer alternative to treat the porcelain surface before bonding.

There was a significant difference between the groups treated with HFA vs. PA with higher SBS values for the HFA-treated groups (9.7±3.8 MPa) in the present study. The mean SBS value for PA groups was 7.5±3.1 MPa, which is still within the clinically accepted range.

However, Bourke and Rock,^9^ Buyuk and Kucukekenci,^1^ and Purmal et al^23^ found that the SBS was similar in groups using HFA and those using PA. Also, in a study by Larmour et al,^11^ conditioning porcelain surfaces with 37% PA resulted in clinically adequate bond strength that is acceptable in the clinical practice and is approximate to that created by the utilization of HFA. Thus, if there is no added advantage of using HFA, one should eliminate it for obvious reasons.

In the present study, CBs had a significantly higher SBS than MBs (9.4±4.3 MPa). The mean SBS value for MBs groups was 7.8±2.6 MPa. These results were similar to those by Elsaka,^24^ who reported that CBs produced greater bond strength than MBs. Also, Ebert et al^25^ found that SBS values were significantly different between metal and CBs.

According to several previous studies, the bond strength of CB appears to be higher than that of MB owing to the stronger bond they have. Also, this could be because of the light transmittance of CBs, which allows superior photo-polymerization and lower stresses at the adhesive‒bracket interface.^5,9,26^

On the other hand, a study by Mehmeti et al^27^ revealed that all-MBs in contrast to CBs produced higher bond strength with all the zirconium surfaces because of their more advanced base surface design or retention mode. However, Abu Alhaija et al^4^ found that both MB and CB produced similar SBS values.

In the present investigation, the recorded ARI score was 4 (<10% of the adhesive remaining on the ceramic surface) in most groups. This suggests a weak adhesion between the porcelain and the adhesive resin. Clinically, failures at the ceramic‒composite interface are favored since ceramic fractures, and extreme smoothening techniques are avoided following debonding. These results are consistent with previous studies by Buyuk and Kucukekenci^1^, Abo alhaija et al,^4^ and Türk et al.^12^

## Conclusion

The three CAD/CAM ceramic materials produced SBS with no significant differences. Etching with HFA significantly increased the bond strength compared to etching with PA. CBs had a significantly higher bond strength than metal ones. The CAD/CAM ceramic material type did not affect the SBS significantly. Both the etching method and the bracket type did affect the SBS values.

## Authors’ Contribution

EAGconceived and designed the work, collected data, contributed to data analysis, and wrote the paper. MSS contributed to work design, supervised the work, critically revised the article, and approved the version to be published. AAEdesigned the work, supervised the work, critically revised the article, finally approved the version to be published.

## Funding

Self-funded.

## Competing Interests

The authors declare no conflict of interests related to the publication of this work.

## Ethics Approval

Not applicable.
